# Hippocampal astrocytes represent navigation space

**DOI:** 10.1371/journal.pbio.3001568

**Published:** 2022-03-08

**Authors:** Xinzhu Yu

**Affiliations:** Department of Molecular and Integrative Physiology, University of Illinois at Urbana-Champaign, Urbana, Illinois, United States of America

## Abstract

Hippocampal place cells, which display location-specific activity, are known to encode spatial information. This Primer explores the implications of a PLOS Biology study showing that hippocampal astrocytes are involved in encoding complementary spatial information, suggesting the existence of glial place cells.

## Teaser

Hippocampal place cells, which display location-specific activity, are known to encode spatial information. A recent study in *PLOS Biology* by Curreli and colleagues shows that hippocampal astrocytes are implicated in encoding complementary spatial information, suggesting the existence of glial place cells.

**Fig 1:**
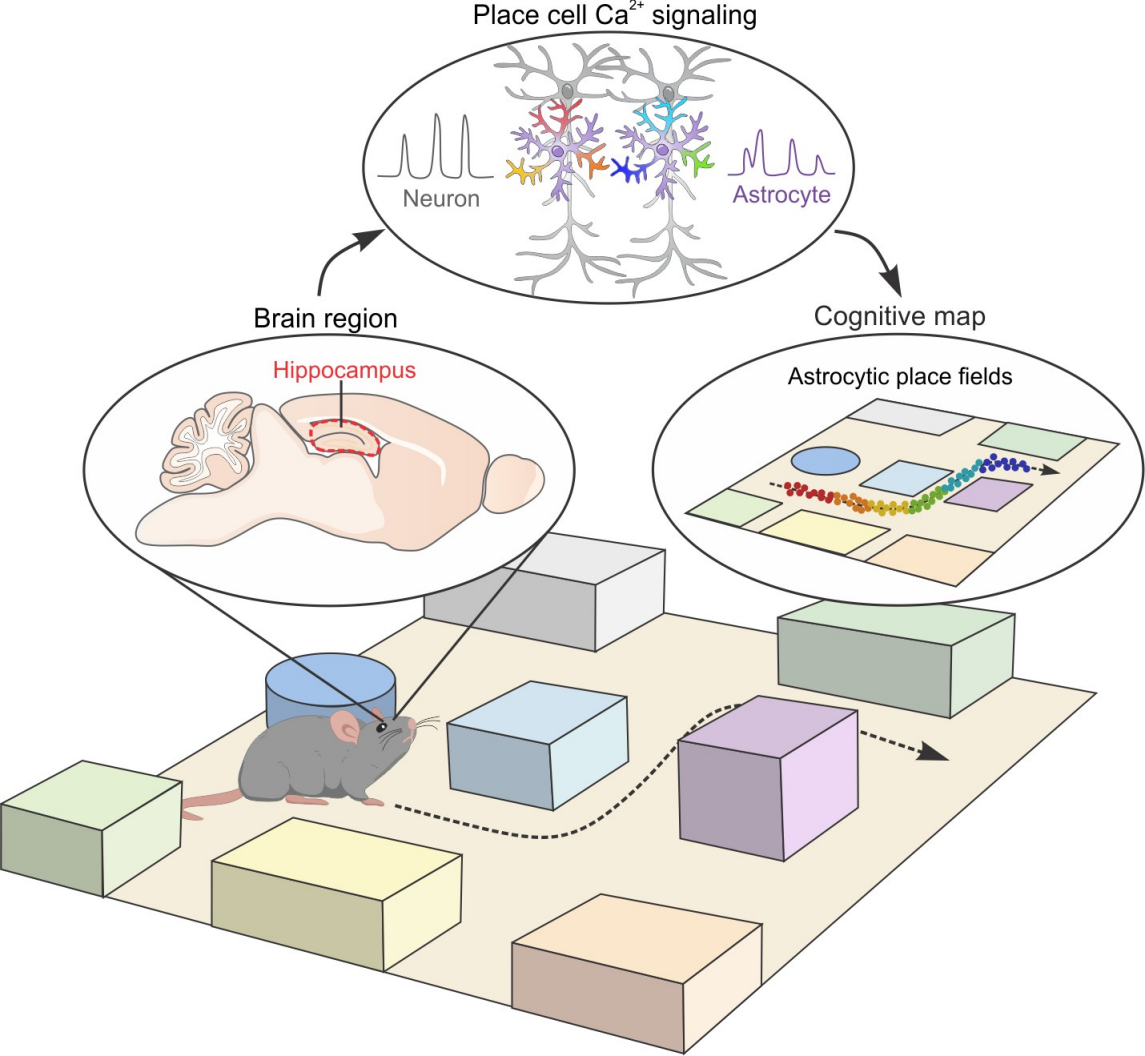
Cognitive map and underlying cellular mechanisms. A cognitive map encodes spatial information and allows navigational planning. The hippocampus is a key structure containing cells responsible for constructing the cognitive map in the mammalian brain. Hippocampal neurons known as place cells become electrically active when the animal travels to specific locations within its environment. Using 2-photon *in vivo* Ca^2+^ imaging, Curreli and colleagues provide evidence that hippocampal astrocytes respond to spatial locations in a virtual reality environment with elevations in their intracellular Ca^2+^ signals. Moreover, the information contained by hippocampal astrocytes is not a passive copy of nearby neuronal information. The tantalizing unanswered question is whether astrocytes are active partners with neurons in generating a spatial cognitive map.

The ability to perceive location and navigate through the space is a cognitive function crucial for both humans and animals. It has been hypothesized that a “cognitive map” is constructed in the mammalian brain based on spatial cues to represent the external environment. This mental map could contribute to the formation of episodic memory that is linked to a specific location and context. In search of the neurobiological basis of the cognitive map, O’Keefe and Nadel first discovered place cells in the rodent hippocampus that become active when the animal travels to a specific location within its environment [1]. Subsequent studies have further revealed additional key elements of the spatial navigation system, including grid cells, head direction cells, and border cells. Collectively, the neuronal circuits of the hippocampal formation are believed to be responsible for constructing a detailed mental model of the external space. Is this the entire story? A research article presented by Curreli and colleagues in the current issue of *PLOS Biology* provides the first *in vivo* evidence that nonneuronal glial cells also display location-dependent activity [2], raising important questions about whether and how glial cells are involved in spatial navigation and cognitive map representation (Fig 1).

As the most numerous type of glial cells, astrocytes are morphologically complex and present throughout the entire central nervous system (CNS). Their existence and close proximity to neurons were depicted by pioneering neuroscientist Santiago Ramón y Cajal more than a century ago. However, our understanding of astrocytes and their contributions to cognition is still in its infancy. This is largely because astrocytes are electrically silent cells that lack mechanisms to generate and propagate action potentials. Therefore, traditional electrophysiological approaches provide little information about astrocyte activity and their function. Instead, astrocytes display dynamic and extensive intracellular Ca^2+^ signals that are believed to be the primary mechanism mediating their communication with other cells [3]. These astrocytic Ca^2+^ signals occur throughout the entire cell (e.g., somata and processes), both spontaneously and in response to neuronal activity. Mounting evidence has implicated astrocytic Ca^2+^ signaling as essential to modulating neuronal function and animal behaviors [4].

Curreli and colleagues recorded intracellular Ca^2+^ signals from hippocampal astrocytes expressing a genetically encoded Ca^2+^ indicator in head-fixed awake mice navigating in a virtual reality environment [2]. Interestingly, the authors found that a population of astrocytes displayed Ca^2+^ responses that were modulated by the position of the mouse, indicative of location-dependent activity that is similar to neuronal place cells. However, in contrast to neuronal place cells, Ca^2+^ signals from the somata and processes belonging to the same astrocytes may respond to different spatial locations, i.e., different regions of a single astrocyte may have distinct place fields.

These are novel and intriguing findings; however, previous studies have demonstrated that astrocyte Ca^2+^ signaling largely reflects the activity of neighboring neurons [5,6]; thus, it is possible that the astrocytes are merely mirroring some spatial information conveyed by nearby neuronal place cells. To account for this possibility, the authors simultaneously recorded Ca^2+^ signals from both astrocytes and neurons in the hippocampus of awake mice navigating in the virtual reality setup. Using an information theory–based analysis and a machine learning approach, the authors showed that the spatial information encoded in astrocyte Ca^2+^ signals is not a simple copy of that stored in surrounding neurons. Specifically, animal’s spatial location was more accurately decoded with Ca^2+^ signal data from neurons and astrocytes combined than from neurons or astrocytes alone, suggesting additional information encoded by hippocampal astrocytes.

These results further raise a conspicuous unanswered question: What spatial information do hippocampal astrocyte Ca^2+^ signals encode? Does astrocyte spatial encoding involve nontrivial computations rather than simple signal summation? Owing to its elaborate morphology, a single hippocampal astrocyte territory is estimated to enclose around 90,000 synapses [7]. Thus, it is possible that hippocampal astrocyte Ca^2+^ signals serve as a tuning factor by integrating diverse neuronal information and subsequently conveying synaptic regulation via ion homeostasis, metabolic support, synaptic formation/removal, and neurotransmitter uptake. Future computational modeling of these potential integrator functions may be fruitful, and experimental manipulations of astrocyte ensembles and their corresponding subcellular Ca^2+^ signals may provide direct evidence.

To understand how the CNS encodes, modifies, stores, and retrieves information, it is necessary to explore the diverse cell populations that comprise the CNS. There is an emerging consensus that the CNS cannot be satisfactorily understood solely as a collection of interacting neurons. One significant missing aspect in our strategy to comprehensively understand the CNS, particularly in the context of disease, is the largely unmet need to understand additional cell types such as astrocytes. This work by Curreli and colleagues has provided a new perspective of astrocytic involvement in the domain previously considered to be uniquely neuronal. In light of their stimulating results, there are many intriguing questions and further directions that remain to be explored. First of all, given astrocytic place fields in a one-dimensional virtual reality environment, how do hippocampal astrocytes behave in realistic environments? In addition to encoding spatial locations, neuronal place cells are fundamental for goal-directed navigation planning, episodic memory storage, and retrieval [8], presumably in conjunction with neurons from other brain regions connected to the hippocampus. Although astrocytes do not project beyond their local territories, can these local actions by astrocytes have a broader effect on large-scale neural circuits by communicating with and modulating other cells in the networks? Furthermore, sequential firing patterns of neuronal place cells activated while navigating are replayed during sleep [9]. This mechanism is proposed to consolidate newly encoded spatial memories. Can hippocampal astrocytes register previous spatial learning by spatially tuned Ca^2+^ signals and consolidate recent memory traces by offline replay of those Ca^2+^ signals? Last, are there equivalent astrocytic subpopulations responding to speed, head direction, and boundaries to construct the complete spatial maps? With many new methods and technological advances on the horizon [10], these questions will be ultimately tackled in the foreseeable future.

## References

[1] O’Keefe J, Nadel L. The hippocampus as a cognitive map. Oxford, New York: Clarendon Press; Oxford University Press; 1978. xiv, p. 570.

[2] Curreli S, Bonato J, Romanzi S, Panzeri S, Fellin T. Complementary encoding of spatial information in hippocampal astrocytes. PLoS Biol. 2022;20(3): e3001530. doi: 10.1371/journal.pbio.3001530 35239646 PMC8893713

[3] Bazargani N, Attwell D. Astrocyte calcium signaling: the third wave. Nat Neurosci. 2016;19(2):182–9. doi: 10.1038/nn.4201 . 26814587

[4] Nagai J, Yu X, Papouin T, Cheong E, Freeman MR, Monk KR, et al. Behaviorally consequential astrocytic regulation of neural circuits. Neuron. 2021;109(4):576–96. Epub 2020 Dec 31. doi: 10.1016/j.neuron.2020.12.008 ; PubMed Central PMCID: PMC7897322. 33385325 PMC7897322

[5] Wang X, Lou N, Xu Q, Tian GF, Peng WG, Han X, et al. Astrocytic Ca2+ signaling evoked by sensory stimulation in vivo. Nat Neurosci. 2006;9(6):816–23. Epub 2006 May 14. doi: 10.1038/nn1703 . 16699507

[6] Schummers J, Yu H, Sur M. Tuned responses of astrocytes and their influence on hemodynamic signals in the visual cortex. Science. 2008;320(5883):1638–43. doi: 10.1126/science.1156120 . 18566287

[7] Chai H, Diaz-Castro B, Shigetomi E, Monte E, Octeau JC, Yu X, et al. Neural Circuit-Specialized Astrocytes: Transcriptomic, Proteomic, Morphological, and Functional Evidence. Neuron. 2017;95(3):531–49.e9. Epub 2017 Jul 14. doi: 10.1016/j.neuron.2017.06.029 ; PubMed Central PMCID: PMC5811312. 28712653 PMC5811312

[8] Robinson NTM, Descamps LAL, Russell LE, Buchholz MO, Bicknell BA, Antonov GK, et al. Targeted Activation of Hippocampal Place Cells Drives Memory-Guided Spatial Behavior. Cell. 2020;183(7):2041–2. doi: 10.1016/j.cell.2020.12.010 ; PubMed Central PMCID: PMC7773032. 33357402 PMC7773032

[9] Atherton LA, Dupret D, Mellor JR. Memory trace replay: the shaping of memory consolidation by neuromodulation. Trends Neurosci. 2015;38(9):560–70. Epub 2015 Aug 11. doi: 10.1016/j.tins.2015.07.004 ; PubMed Central PMCID: PMC4712256. 26275935 PMC4712256

[10] Yu X, Nagai J, Khakh BS. Improved tools to study astrocytes. Nat Rev Neurosci. 2020;21(3):121–38. Epub 2020 Feb 10. doi: 10.1038/s41583-020-0264-8 . 32042146

